# Ecology and morphological variations in wings of *Phlebotomus ariasi* (Diptera: Psychodidae) in the region of Roquedur (Gard, France): a geometric morphometrics approach

**DOI:** 10.1186/s13071-016-1872-z

**Published:** 2016-11-14

**Authors:** Jorian Prudhomme, Cécile Cassan, Mallorie Hide, Céline Toty, Nil Rahola, Baptiste Vergnes, Jean-Pierre Dujardin, Bulent Alten, Denis Sereno, Anne-Laure Bañuls

**Affiliations:** 1 UMR MIVEGEC (IRD 224 - CNRS 5290 - Université de Montpellier), 911 avenue Agropolis, Montpellier, F34394 France; 2 UMR INTERTRYP (IRD - CIRAD 177), Centre IRD, Montpellier, F34394 France; 3 Department of Biology, Ecology Section, Faculty of Science, Hacettepe University, HU-ESRL-VERG Laboratories, Beytepe, Ankara 0680 Turkey

**Keywords:** Sand fly, Southern France, Geometric morphometry, *Phlebotomus ariasi*, Phenotypic plasticity

## Abstract

**Background:**

*Phlebotomus ariasi* Tonnoir, 1921, is the predominant sand fly species in the Cevennes region and a proven vector of *Leishmania infantum*, which is the main pathogen of visceral and canine leishmaniasis in the south of France. Even if this species is widely present in Western Mediterranean countries, its biology and ecology remain poorly known. The main goals of this work are to investigate the phenotypic variation of *P. ariasi* at a local scale in a region characterized by climatic and environmental fluctuations, and to determine if slope and altitude could affect the sand fly phenotypes.

**Results:**

Sand flies were captured along a 14 km-long transect in 2011 from May to October. At the same time, environmental data such as altitude and slope were also collected. Morphological analysis of *P. ariasi* wings was performed by a geometric morphometrics approach. We found morphological variation among local populations of *P. ariasi*. Strong shape and size variations were observed in the course of the season (particularly in June and July) for both genders. During June, we highlighted differences in wing phenotypes according to altitude for both sexes and to slope and station for females.

**Conclusions:**

The phenotypic variations observed in *P. ariasi* along the studied transect indicated these populations are subjected to environmental pressures. Nevertheless, it seems that sand flies are more sensitive to extrinsic factors in June and July, suggesting a phenotypic plasticity.

## Background

*Phlebotomus ariasi* Tonnoir, 1921 is the predominant sand fly species in the Cevennes region [1] and one of the two proven vectors of leishmaniasis caused by *Leishmania infantum* in the south of France, with *Phlebotomus perniciosus* Newstead, 1911 [2]. The resting sites of *P. ariasi* are found in houses, animal sheds, caves and holes in walls (“barbacanes”), near roads or in villages. This species is active during dusk and night, present during summer in temperate regions, abundant in peri-urban and rural environments and it often lives close to human and domestic animal populations [3]. This species is widely present in Western Mediterranean countries [4–6].

Currently, biology and ecology of *P. ariasi* remain poorly known. During the past 10 years, the risk of emergence or re-emergence of leishmaniasis has increased in France [7]. The current expansion of the distribution of this disease underlines the need to increase knowledge on this vector. However, only few studies have been performed to understand the population structure of this species and they were based on the analysis of cuticular hydrocarbons [8], isoenzymes [9], random amplified polymorphic DNA [10], sequences of the mitochondrial cytochrome *b* gene [5] and microsatellite markers [11]. Until now, no study using a geometric morphometrics approach has been done to investigate phenotypic variations of *P. ariasi* in France. Significant morphometric wing shape variations were found in other sand fly species within populations originating from various Mediterranean regions, forming clusters or latitudinal clines in other species, such as *P. papatasi* [12, 13] and *P. sergenti* [14, 15]. These studies highlighted the existence of local phenotypic variations linked to environmental factors.

The main goals of this work were to investigate phenotypic variations of *P. ariasi* populations collected at a local scale in a region characterized by climatic and environmental fluctuations, and to determine if slope and altitude could affect sand fly phenotypes.

## Methods

### Study area

**Fig. 1:**
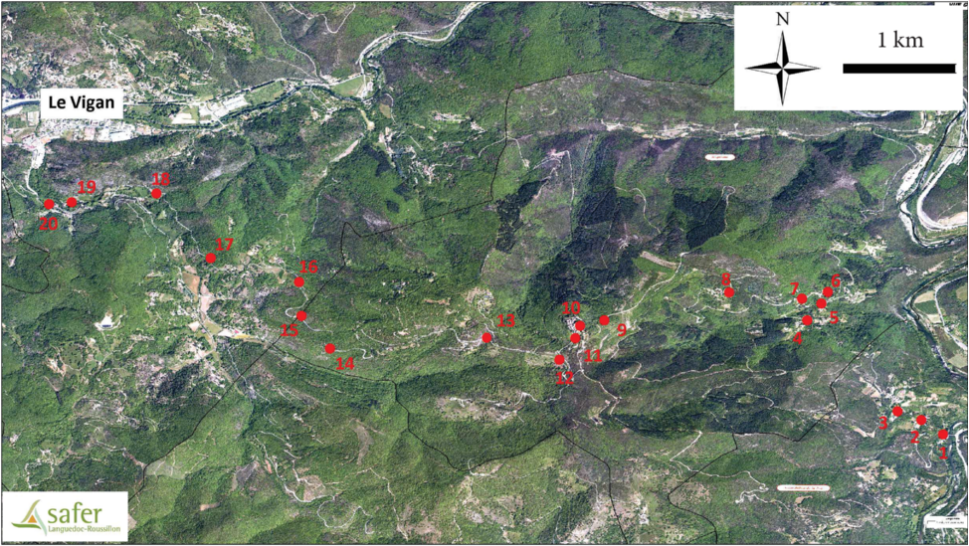
Map of the study area showing the main sites sampled. Red circles indicate the sampling localities

The field study was performed in the south of France, on the hill “le massif de l’Oiselette” located between two valleys: “Hérault” (Ganges, Hérault) and “Arre” (Le Vigan, Gard). Sand flies were collected along a 14 km transect between Saint Julien de la Nef and le Vigan, including Roquedur-le-Haut (at 601 m above sea level) (Fig. 1). Twenty stations were selected along this transect (Table 1 and Fig. 1). This region is subject to the Mediterranean sub-humid climate [16] and characterized by the presence of “Garrigue” species such as *Quercus ilex* and *Quercus pubescens*.

**Table 1:** Sampling stations in the study area

Station number	Coordinates		Altitude (m)	Biotope	Traps
	North	East			
ST01	43.96548	3.686828	175	Hamlet	CDC + ST
ST02	43.96663	3.685075	228	Hamlet	ST
ST03	43.96733	3.683149	244	Hamlet	CDC + ST
ST04	43.97462	3.675871	321	Hamlet/Hutch	CDC + ST
ST05	43.97595	3.677038	322	Rural	ST
ST06	43.97687	3.677551	341	Kennel	CDC + ST
ST07	43.97632	3.675457	354	Hamlet	CDC + ST
ST08	43.97683	3.669611	443	Hamlet	ST
ST09	43.9746	3.659549	586	Poultry	CDC
ST10	43.97416	3.657616	606	Poultry	CDC
ST11	43.97317	3.657214	603	Hamlet	CDC + ST
ST12	43.97144	3.655944	573	Rural	ST
ST13	43.97321	3.650088	539	Rural	CDC + ST
ST14	43.97235	3.637441	417	Rural	ST
ST15	43.97495	3.635153	397	Rural	CDC + ST
ST16	43.97765	3.634949	362	Rural	ST
ST17	43.97961	3.627845	343	Rural	CDC + ST
ST18	43.98478	3.623463	282	Rural	CDC + ST
ST19	43.98409	3.616661	255	Hamlet	CDC + ST
ST20	43.98392	3.614822	245	Hamlet/Sheep barn	CDC + ST

*CDC* CDC miniature light traps, *ST* Sticky Traps

This geographical area is known to be an endemic area for human and canine leishmaniasis caused by *L. infantum* [1]. Various domestic animals that are potential hosts for sand flies were present throughout the transect, such as chicken, sheep, ducks, geese, horses, rabbits, cats and dogs. Furthermore, some stations were located in semi-rural areas and thus various wild animals could be also hosts for sand flies [17].

To test the effect of environmental factors on the sand fly wing size and shape, the stations were organized according to the slope and altitude. Therefore, stations were grouped into two slope groups: Southeast (SE) and Northwest (NW), and five altitudinal groups: 0 (100–200 m), 1 (200–300 m), 2 (300–400 m), 3 (400–500 m), 4 (> 500 m) (Table 2).

**Table 2:** Number of *Phlebotomus ariasi* wings used by station for the geometric morphometric analysis

Station	Slope	Altitude groups	Wing number by month											
			May		June		July		August		September		Total	
			♂	♀	♂	♀	♂	♀	♂	♀	♂	♀	♂	♀
ST01	SE	0^a^	0	0	0	0	0	0	0	0	0	0	0	0
ST02	SE	0	0	0	0	0	0	0	0	0	0	0	0	0
ST03	SE	1^b^	0	0	4	4	2	6	0	0	0	0	6	10
ST04	SE	2^c^	0	0	1	14	6	0	0	0	0	0	7	14
ST05	SE	2	0	0	8	0	0	0	0	0	0	0	8	0
ST06	SE	2	0	0	6	14	4	16	8	0	0	1	18	31
ST07	SE	3^d^	0	0	0	0	0	0	0	0	0	0	0	0
ST08	SE	4^e^	0	0	0	0	0	0	0	0	0	0	0	0
ST09	SE	4	0	0	9	9	2	5	0	0	0	0	11	14
ST10	SE	4	0	0	0	0	0	2	0	1	0	0	0	3
ST11	NW	4	1	0	9	23	3	19	5	2	3	0	21	44
ST12	NW	4	4	0	6	3	0	0	0	0	0	0	10	3
ST13	NW	4	0	0	7	0	0	1	1	1	4	1	12	3
ST14	NW	3	3	0	12	0	2	0	0	0	2	0	19	0
ST15	NW	2	0	0	5	0	2	0	1	0	0	0	8	0
ST16	NW	2	1	0	12	0	0	0	0	0	0	0	13	0
ST17	NW	2	0	0	0	2	0	4	0	0	0	0	0	6
ST18	NW	1	0	0	8	4	5	9	2	0	1	0	16	13
ST19	NW	1	0	0	0	4	8	4	0	0	0	0	8	8
ST20	NW	1	0	0	27	32	2	7	0	0	0	0	29	39
Total			9	0	114	109	36	73	17	4	10	2	186	188

*Abbreviations*: *SE* southeast, *NW* northwest, ♂ male, ♀ female

^a^100–200 m altitude

^b^200–300 m altitude

^c^300–400 m altitude

^d^400–500 m altitude

^e^ > 500 m altitude

### Sand fly collection and identification

Sand fly collections were performed monthly between May and November 2011 with CDC miniature light traps (John W. Hock Co. FL, USA) and flight interception traps (20 × 20 cm white paper cover with castor oil) [18]. Along this transect, 20 stations were selected. In 14 sampling sites (Table 1), one or two light traps were set up for two nights (inside and/or outside of houses, animal barns, etc.), operating between 18:00 pm and 08:00 am. A total of 105 light traps were set during 210 nights of trapping. In 17 stations (Table 1), a total of 3,589 sticky traps were used and a mean of 189 sticky traps by station were placed in various biotopes, inside and around human dwelling and animal housing, close to the vegetation and crevices in the walls. The sticky traps were settled up in the sampling sites for 2 consecutive days.

At the end of the sampling period, collected specimens were transferred individually into 1.5 ml Eppendorf tubes with 96% ethanol and labeled accordingly. Prior to mounting, heads, genitalia and wings of the sand flies were removed. The heads and genitalia were cleared in Marc-André solution (chloral hydrate/acetic acid) and mounted in chloral gum [3]. Specimen identification was individually verified based on the morphology of the pharynges and/or the male genitalia or female spermathecae, using the keys of Abonnenc [3], Lewis [6] and Killick-Kendrick et al. [19]. From the identifications, we selected the wings of the *P. ariasi* specimens, which were in the majority of our sample.

### Wing preparation

A total of 374 specimens of *P. ariasi* were used for the geometric morphometrics analysis (186 males and 188 females) (Table 2). Rohlf et al. [20] suggest using only one side for the pair of organs or limbs to avoid asymmetry bias between the two sides. In this study, only right wings of specimens were used. They were stained using the method previously described in Prudhomme et al. [13] and then, mounted in Euparal on labeled slides. The wing slides were photographed using a Leica Z16 APOA stereoscopic zoom dissection microscope with DFC 425 digital camera system, digitized, and archived.

### Morphometric analysis

**Fig. 2:**
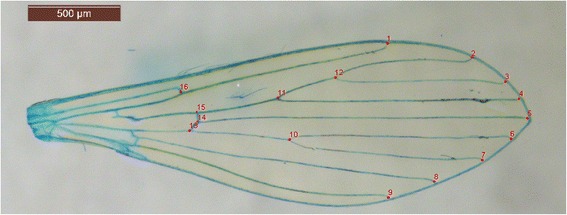
Location of the 16 wing landmarks used in the morphometric analysis of *Phlebotomus ariasi*

Pictures were first entered into tps-Util 1.60 [21]. Then, 16 landmarks were used for the analysis following the method of Rohlf et al. [20] with tpsDIG2 2.18 software [22] (Fig. 2). Landmarks are located at the intersections of wing veins with the wing margin and at the intersections of cross veins with major veins (Fig. 2). The morphometric analyses as well as graphical outputs were performed using various modules of the CLIC package [23]. The centroid sizes were analyzed as a size estimator using a nonparametric Wilcoxon-Mann-Whitney test or Kruskal-Wallis test followed by a *post-hoc* test using Mann-Whitney tests with Bonferroni correction, using the statistical package R, version 3.1.2 [24]. Centroid size is the square root of the sum of squared distances of a set of landmarks from their centroid, i.e. the square root of the sum of the variances of the landmarks about that centroid in x- and y- directions [25]. The landmark configurations were scaled, translated, and rotated against the consensus configuration by the GLS Procrustes superimposition method [25–28] in order to produce shape variables (partial warps, PW). The principal components (based on the partial warps) [25] were used to compare population samples. To assess the degree of similarity between populations, pairwise Mahalanobis distances between populations were calculated using CLIC software [23] and tested by nonparametric permutation tests with 1,000 iterations each. These distances were also used to perform a simple reclassification test for each individual. The percentage of correctly assigned individuals to the corresponding group was assessed. Finally, residual allometry (contribution of size to wing shape) was estimated by multivariate regression of PW on size.

## Results

### Sexual dimorphism

**Fig. 3:**
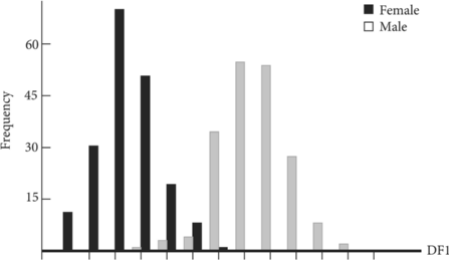
Distribution of individuals along the first discriminant factor (DF1) of shape analysis by gender

**Fig. 4:**
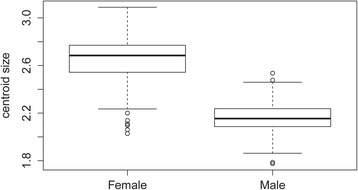
Mean, standard deviation and error of centroid wing sizes for each sex

**Fig. 5:**
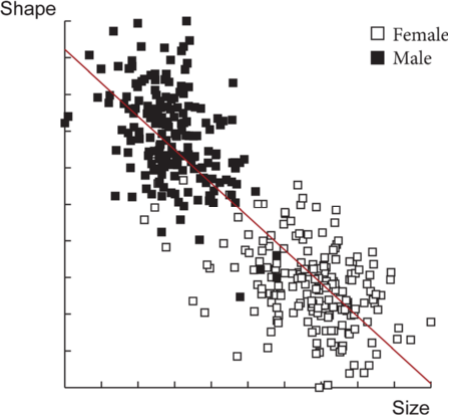
Regression of first discriminant factor on centroid size. Vertical axis: first discriminant factor, representing 100% of the total discrimination; Horizontal axis: centroid size of the wing. The analysis was based on the partial warps. Regression line is shown

The wing shape showed statistically significant differences between males and females (Fig. 3). Mahalanobis distances were significantly different between both sexes (Adjusted *P*-value < 0.0001). Moreover, simple reclassification scores to the sexes were equal to 96% for males and 93% for females, supporting the observed differentiation between the two groups. Centroid sizes were used as a measure of the overall wing size differences among populations. The wing size linked to the gender was found to be significantly different (Kruskal-Wallis test : *χ* ^2^ = 253.4024, *df* = 1, *P* < 0.0001), with females displaying larger wings than males (Fig. 4). The contribution of size to wing shape differentiation was 72% (Fig. 5).

Since a phenotypic difference between males and females was observed, the subsequent analyses were performed separately for each sex.

### Differentiation by month

**Fig. 6:**
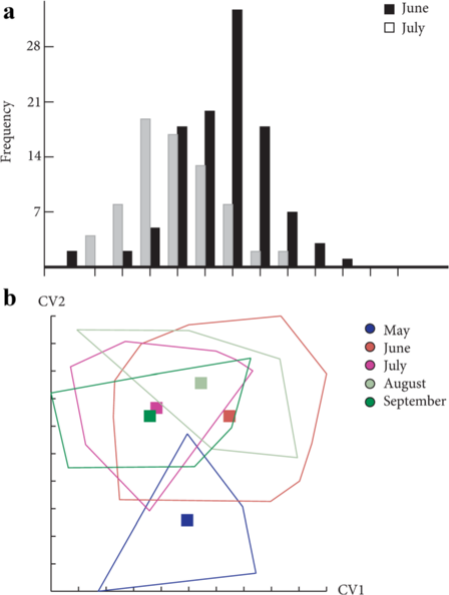
Distribution of the individuals along the discriminant factors of shape analysis in June and July. **a** Distribution of the female individuals along the discriminant factor. **b** Distribution of the male individuals along the first two discriminant factors. The analysis was based on the partial warps. *Abbreviations*: CV1, discriminant factor 1; CV2, discriminant factor 2

Mahalanobis distances, used to investigate wing shape variations, were significantly different between the months; between June-July for females (Ajusted *P*-value < 0.0001) and between June-July and July-August for males (Ajusted *P*-value < 0.05 after Bonferroni correction; eight components, 85.53% of total shape variance) (Fig. 6). For females, it was not possible to test shape differentiation for the other months due to the small number of specimens (Table 2). Indeed, for the discriminant analyses, the sampling size is a limiting factor; the low number of principal components does not allow explaining the variance of the dataset. Moreover, simple reclassification scores to the month groups were on average equal to 73.6% for males (58–88%) and 81% for females (80–82%), supporting the differentiation observed between samples.

**Fig. 7:**
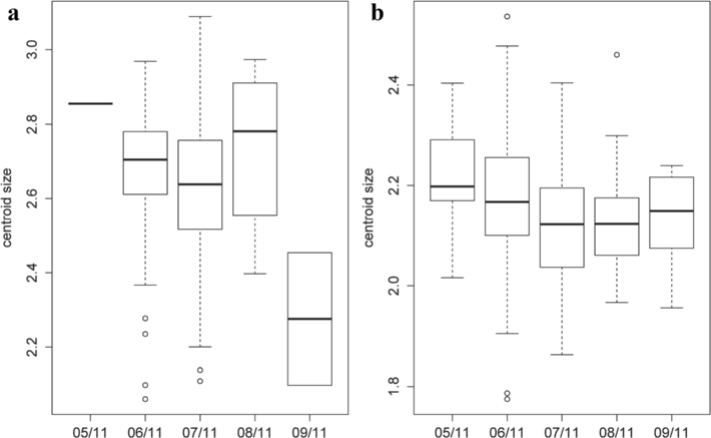
Mean, standard deviation and error of centroid wing sizes by month. **a** Females. **b** Males

**Fig. 8:**
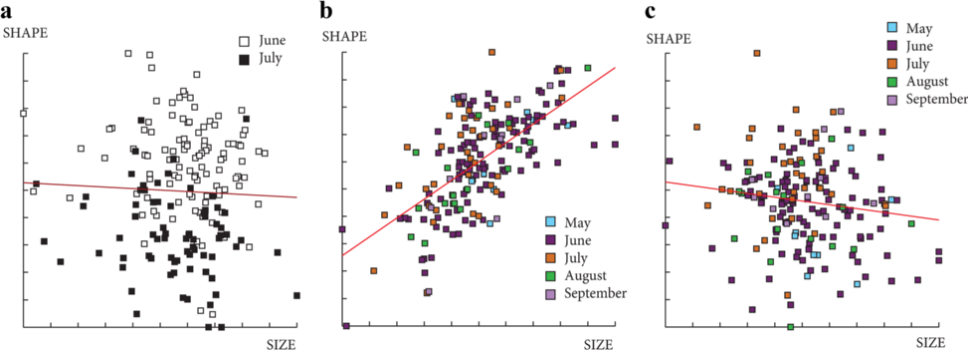
Regression of the discriminant factors on centroid size by months. **a** Regression of the first discriminant factor for females in June and July. **b**, **c** Regression of the two first discriminant factors for males by month. Horizontal axis: centroid size of the wing; Vertical axis (**a**): discriminant factor 1, representing 100% of the total discrimination; Vertical axis (**b** and **c**): discriminant factors 1 and 2, representing 24% and 17% of the total discrimination, respectively. This regression was based on the partial warps. Regression line is shown. Squares indicate individual sand flies

Concerning wing size, a Kruskal-Wallis test revealed a significant effect of month on the centroid size (*χ* ^2^ = 11.6239, *df* = 4, *P* = 0.02038) for females. The *post-hoc* test showed significant differences between June and July (*P* = 0.03741), between June and September (*P* = 0.02588) and between July and September (*P* = 0.03678) (Fig. 7a). For males, no size difference between months was ascertained (*χ* ^2^ = 8.5591, *df* = 4, *P* = 0.07312) (Fig. 7b). The contribution of size to wing shape differentiation was 0% for females and for males 43% and 2% (Discriminant factors 1 and 2, respectively) (Fig. 8).

These results showed a strong phenotypic structuring between months, in particular between June and July for both sexes. Since a phenotypic difference between these two last months was observed, the following analyses performed to determine the possible effects of slope, altitude and station were realized for females and males in June and July separately. It was not possible to test for the other months due to a too small number of specimens (Table 2).

### Differentiation by slope

As detailed above to determine the possible effect of slope on *P. ariasi* phenotype, we performed analysis on wings considering each sex and the two months, June and July, separately (see Table 2).

**Fig. 9:**
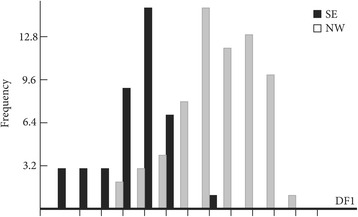
Distribution of female individuals along the first discriminant factor of shape analysis according to slopes. This distribution was based on the partial warps in June. *Abbreviations*: SE, southeast slope; NW, northwest slope

Mahalanobis distances to study wing shape were significantly different between the slopes (Ajusted *P*-value < 0.01) only for females in June (Fig. 9). Moreover, simple reclassification scores to the slope groups were on average equal to 95% (80–90%), supporting the observed differentiation between samples. However, no significant difference was observed between the slopes for the females in July (Ajusted *P*-value > 0.05) and for the males for any month (Ajusted *P*-values > 0.05; 13 components, 96.5% of total shape variance) (data not shown).

Furthermore, the Kruskal-Wallis test to study wing size revealed also no significant effect of slopes in June and July (*χ* ^2^ = 1.0064, *df* = 1, *P* = 0.3158; *χ* ^2^ = 0.4636, *df* = 1, *P* = 0.4959, respectively) for females and also for males (*χ* ^2^ = 0.4915, *df* = 1, *P* = 0.4833; *χ* ^2^ = 0.6771, *df* = 1, *P* = 0.4106) (data not shown).

**Fig. 10:**
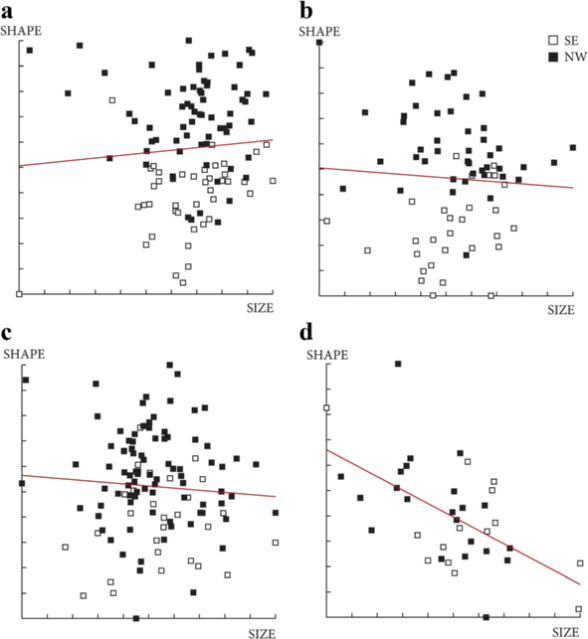
Regression of first discriminant factor of shape analysis on centroid size from females (**a**, **b**) and males (**c**, **d**). Horizontal axis: centroid size of the wing; Vertical axis: first discriminant factor, representing 100% of the total discrimination. This regression was based on the partial warps in June (a, c) and July (b, d). Regression line is shown. Signs indicate each individual. *Abbreviations*: SE, southeast slope; NW, northwest slope

The contribution of size to wing shape differentiation was 0% for females for both months and 0 and 32% for males in June and July, respectively (Fig. 10).

### Differentiation by altitude

**Fig. 11:**
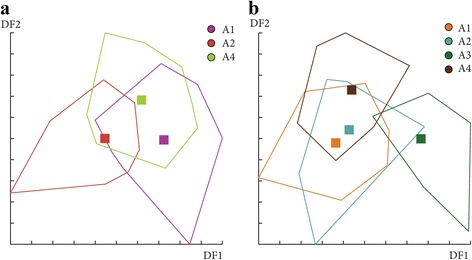
Distribution of the individuals along the first two discriminant factors of shape analysis by altitude groups for females (**a**) and males (**b**). This distribution was based on the partial warps in June. Horizontal axis: discriminant factor 1; Vertical axis: discriminant factor 2. Altitude groups: 1 (200–300 m), 2 (300–400 m), 3 (400–500 m), 4 (> 500)

The effect of altitude on *P. ariasi* phenotype was tested for females and males in June and July separately (see Table 2). In June, Mahalanobis distances used to study wing shape were significantly different between the altitudinal groups 1 and 2 for females (Ajusted *P-*value < 0.01667) and between group 3 and all the other groups for males (Ajusted *P-*value < 0.00833, 11 components, 93.2% of total shape variance) (Fig. 11). Moreover, simple reclassification scores to the altitudinal groups were on average equal to 73% (65–80%) for females and 53% (43–75%) for males, supporting the observed differentiation between samples. However, in July, no significant difference was observed between the groups for females and males (Ajusted *P-*value > 0.01667, 19 components, 99.2% of total shape variance and Ajusted *P-*value > 0.01667, 4 components, 71.3% of total shape variance, respectively) (data not shown). To realize the analyses with males in July, the group 4 had to be removed because of a low number of specimens (*n* = 2).

**Fig. 12:**
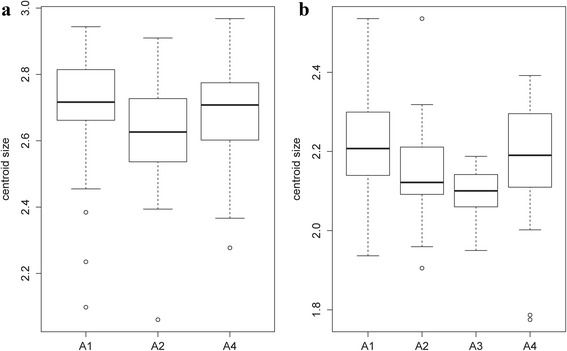
Mean, standard deviation, and error of centroid wing sizes by altitude groups in June for females (**a**) and males (**b**). Altitude groups: 1 (200–300 m), 2 (300–400 m), 3 (400–500 m), 4 (> 500 m)

Concerning wing size, in June, a significant effect of altitude on centroid size was observed for females (*χ* ^2^ = 7.0282, *df* = 2, *P* = 0.0298), the *post-hoc* test showed significant differences between groups 1 and 2 (*P* = 0.022). For males, the difference was strongly significant between groups 1 and 3 (*χ* ^2^ = 12.4331, *df* = 3, *P* = 0.006) with a *P*-value of 0.0076 for the *post-hoc* test (Fig. 12). Conversely, no significant effect of altitude was found on the centroid sizes in July for both sexes (*χ* ^2^ = 4.9144, *df* = 2, *P* = 0.0857; *χ* ^2^ = 0.4848, *df* = 3, *P* = 0.9222, for females and males respectively) (data not shown).

**Fig. 13:**
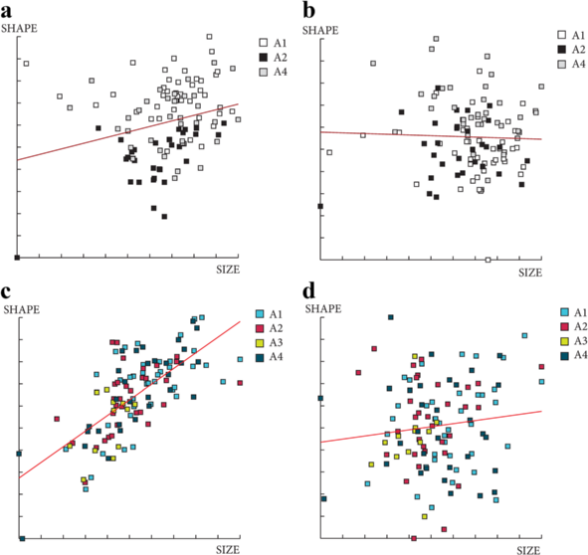
Regression of first discriminant factor on centroid size by altitude groups in June for females (**a**, **b**) and males, (**c**, **d**). Horizontal axis: centroid size of the wing. Vertical axis: discriminant factor 1 (**a**, **c**) or discriminant factor 2 (**b**, **d**). The regression was based on the partial warps. Regression line is shown. Squares indicate individual sand flies. Altitude groups: 1 (200–300 m), 2 (300–400 m), 3 (400–500 m), 4 (> 500)

The contribution of size to wing shape differentiation was 7 and 0% for females and 48 and 1% for males in June (Fig. 13) and 37 and 14% for females and 36 and 0% for males in July (data not shown).

### Phenotypic differentiation by station

In order to observe a possible effect of stations on wing phenotype, several analyses were realized by sex in June and July. Due to low number of individuals in certain stations, we performed a serial of discriminant analyses considering first all the stations and then excluding stations with a low number of individuals in order to increase the number of principal components and thus the variance explained (Table 3).

**Table 3:** Number of *Phlebotomus ariasi* wings used by station for the geometric morphometric analysis

	Females in June		Females in July			Males in June			Males in July		
NC	3	8	3	4	5	3	4	5	2	3	4
V (%)	59.29	87.67	55.32	64.88	70.04	54.91	63.67	71.24	54.65	67.69	75.66
ST03	4	–	6	6	6	4	–	–	–	–	–
ST04	14	14	–	–	–	–	–	–	6	6	6
ST05	–	–	–	–	–	8	8	8	4	4	–
ST06	14	14	16	16	16	6	6	6	–	–	–
ST09	9	9	5	5	–	9	9	9	–	–	–
ST10	–	–	–	–	–	–	–	–	–	–	–
ST11	23	23	19	19	19	9	9	9	3	–	–
ST12	–	–	–	–	–	6	6	6	–	–	–
ST13	–	–	–	–	–	7	7	7	–	–	–
ST14	–	–	–	–	–	12	12	12	–	–	–
ST15	–	–	–	–	–	5	5	–	–	–	–
ST16	–	–	–	–	–	12	12	12	–	–	–
ST17	–	–	4	–	–	–	–	–	–	–	–
ST18	4	–	9	9	9	8	8	8	5	5	5
ST19	4	–	4	–	–	–	–	–	8	8	8
ST20	32	32	7	7	7	27	27	27	–	–	–

*Abbreviations*: *NC* number of components included in the analysis, *ST* station, *V* percentage of shape variance explained by the components included in the analysis

**Fig. 14:**
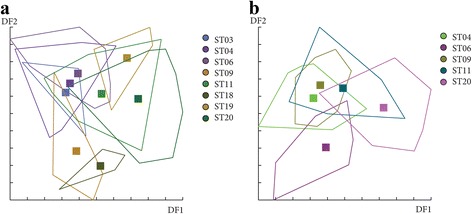
Distribution of the individuals along the first two discriminant factors of shape analysis by station. The analysis was based on the partial warps on female individuals in June with 3 (**a**) and 8 components (**b**). Horizontal axis: discriminant factor 1; Vertical axis: discriminant factor 2

For females in June, Mahalonobis distances were not significantly different between stations for the first analysis based on the 3 principal components included (data not shown), but significant between ST19 and ST20 for the second analysis based on 8 principal components (Table 4 and Fig. 14). In July, even if Canonical variate analysis (CVA) showed some grouping by station (data not shown), Mahalonobis distances were not significantly different between stations for all analyses (Table 4). For males, no significant difference was observed between stations for June and July (data not shown). The results of the shape analyses and the contribution of size to wing shape differentiation are synthesized in Table 4.

**Table 4:** Synthesis of the Mahalonobis distances results

Analysis	NC	V (%)	Ajusted *P*-value	Allometry^a^ (%)	
				1	2
Females in June	3	59.29	> 0.00179	26	1
	8	87.67	< 0.00500^b^	25	22
Females in July	3	55.32	> 0.00179	38	13
	4	64.88	> 0.00333	41	15
	5	70.04	> 0.00500	33	23
Males in June	3	54.91	> 0.00076	48	1
	4	63.67	> 0.00091	49	0
	5	71.24	> 0.00111	48	1
Males in July	2	54.65	> 0.00500	36	0
	3	67.69	> 0.00833	35	1
	4	75.66	> 0.01667	26	8

*Abbreviations*: *NC* number of components included in the analysis, *V* percentage of shape variance explained by the components included in the analysis

^a^Percentage of size contribution to wing shape differentiation for the first component (1) and the second one (2)

^b^Significant effects

Kruskal-Wallis tests used to study wing size revealed no significant effect of station on centroid size in June (*χ* ^2^ = 12.4043, *df* = 9, *P* = 0.1915; *χ* ^2^ = 18.3754, *df* = 11, *P* = 0.07327) and in July (*χ* ^2^ = 12.5229, *df* = 8, *P* = 0.1294; *χ* ^2^ = 11.6, *df* = 9, *P* = 0.2368) for females and males, respectively.

## Discussion

### Sexual dimorphism

Our results showed significant differences in wing shape and size between males and females; females have larger wings. Sexual dimorphism is widely observed in many insects such as *Mansonia* [29] and *Aedes* mosquitoes [30], *Anopheles*, *Culex* and *Ochlerotatus* mosquitoes [31], *Drosophila melanogaster* [32] and *D. subobscura* [33]. Thin-plate spline deformation analyses showed that the deformation was mostly present on the medial part of the wings. The medial of the wings of flying insects is known to play an essential role in flying capability [34]. Because of blood meal necessity, the female sand flies might need a greater flying capacity compared to males. This could explain the wing differences between them. A previous study based on a mark-release-recapture method showed that *P. ariasi* females present a dispersion ability of more than 1 km [35]. It would be interesting to measure the dispersion capacity of males in order to test this hypothesis.

### Wing phenotype differentiation by month

We observed a difference of wing shape between June and July for both sexes and a difference of size only for females (smaller in July). It was not possible to make any conclusion for the other months (May, August and September) due to the low number of individuals captured. These differences observed between June and July may be due to environmental variations as it was observed in previous study [36]. The individuals captured in June and July have certainly emerged at the end of May and June, respectively. Major temperature and relative humidity differences were observed between these 2 months. Moreover, the temperature fluctuations between night and day are greater in May than in June or July. Even if the exact conditions are not well known, the climatic parameters influence the entry into diapause and the exit and also the larvae development of insects [37]. *Phlebotomus ariasi*, as most of northern insect species, presents a period of diapause (at the forth-instar larval stage) in order to survive over the winter and postpone the reproduction until favorable conditions [38]. The climatic differences between months, at the end and/or at the beginning of the sand fly season may be responsible of differences in the development of the larvae before and/or after the diapause [39]. Indeed, the differences in larvae development could impact the diapausing stage and/or adult phenotype.

### Environmental factors and phenotypic variation in June

The data analyses revealed shape and size differences by altitude for males and females in June suggesting differential environmental and climatic pressures according to altitude. Previous studies highlighted the impact of altitude and temperature (two correlated parameters) on sand fly biology [12, 40, 41]. The size of adults is largely influenced by temperature, with larger adults found at low temperatures and smaller adults at high temperatures [12]. It is known that larvae development is influenced by temperature conditions [12, 42]; a high temperature will produce a rapid development and thus small individuals. Assuming that wing size reflects the size of specimens, centroid size should predict larger individuals at lower temperatures and thus at higher altitudes [43]. However, the size differences observed by altitudinal groups did not show significantly larger wings at higher altitudes. This correlation may not be observed in this study because of too small altitudinal differences and thus temperature fluctuations between sampling sites.

We also found a significant spatial differentiation in June for females by slope and station. The absence of spatial differentiation observed in males, conversely to females could be a consequence of stronger sex-specific selection pressures such as the host availability for blood meal in the sampled stations. The strong sexual dimorphism observed also support the existence of sex-specific selection pressures (see above). Nevertheless, the reasons of differential shape and size between sexes have not been well explored [44].

## Conclusions

In conclusion, this study showed phenotypic variations among local populations of *P. ariasi* of different types: sexual dimorphism, shape and size variations between months (June and July in particular) and different wing phenotypes according to altitude for both sexes and by slope and station for females.

The degree of phenotypic variation observed in *P. ariasi* populations seems to reflect the local environmental fluctuations in terms of climatic but also biotic characteristics to which these populations are subjected. These data underline the plasticity and the capacity of adaptation of these insects even at a sympatric level.

These wing variations may have an effect on the biology of *P. ariasi* in terms of dispersion, fitness and transmission of pathogens such as *Leishmania*. Indeed, some effects were recently demonstrated for other insects such as relationships between wing shape and reproductive mode of *Lysiphlebus fabarum* group (Hymenoptera) [45] or enhancement of flight performance by genetic manipulation of wing shape in Drosophila [46]. Further studies are necessary to investigate the impact of wing variations on sand fly biological and ecological traits.

## Abbreviations

CDC: Communicable Disease Center miniature light trap
CVA: canonical variate analysis
NC: number of components included in the analysis
NW: northwest
SE: southeast
ST: sticky trap
V: percentage of shape variance explained by the components included in the analysis
